# Cats and Carbohydrates: The Carnivore Fantasy?

**DOI:** 10.3390/vetsci4040055

**Published:** 2017-11-15

**Authors:** Adronie Verbrugghe, Myriam Hesta

**Affiliations:** 1 Department of Clinical Studies, Ontario Veterinary College, University of Guelph, 50 Stone Road E, Guelph N1G 2W1 ON, Canada; 2 Laboratory of Animal Nutrition, Faculty of Veterinary Medicine, Ghent University, Heidestraat 19, Merelbeke B-9820, Belgium; Myriam.Hesta@ugent.be

**Keywords:** carbohydrates, carnivore, diabetes mellitus, feline, glucose metabolism, hyperglycaemia, macronutrient selection, obesity

## Abstract

The domestic cat’s wild ancestors are obligate carnivores that consume prey containing only minimal amounts of carbohydrates. Evolutionary events adapted the cat’s metabolism and physiology to this diet strictly composed of animal tissues and led to unique digestive and metabolic peculiarities of carbohydrate metabolism. The domestic cat still closely resembles its wild ancestor. Although the carnivore connection of domestic cats is well recognised, little is known about the precise nutrient profile to which the digestive physiology and metabolism of the cat have adapted throughout evolution. Moreover, studies show that domestic cats balance macronutrient intake by selecting low-carbohydrate foods. The fact that cats evolved consuming low-carbohydrate prey has led to speculations that high-carbohydrate diets could be detrimental for a cat’s health. More specifically, it has been suggested that excess carbohydrates could lead to feline obesity and diabetes mellitus. Additionally, the chances for remission of diabetes mellitus are higher in cats that consume a low-carbohydrate diet. This literature review will summarise current carbohydrate knowledge pertaining to digestion, absorption and metabolism of carbohydrates, food selection and macronutrient balancing in healthy, obese and diabetic cats, as well as the role of carbohydrates in prevention and treatment of obesity and diabetes mellitus.

## 1. Introduction

The domestic cat’s wild ancestors (*Felis silvestris*) are known to be obligate carnivores. From a nutritional perspective, this means that in their natural habitat cats consume small prey, including rodents and birds, which are high in protein, moderate in fat and include only minimal carbohydrates. Data from dietary habits of feral cats combined with compositional data of the consumed prey species revealed a typical diet containing a crude protein, crude fat and nitrogen-free extract (NFE) content of 52%, 46% and 2% of metabolisable energy (%ME), respectively [1]. These dietary habits have led to specific and unique nutritional requirements. In cats, the dietary requirements for protein, arginine, taurine, methionine and cystine, arachidonic acid, niacin, pyridoxine, vitamin A and vitamin D are greater than for omnivores due to metabolic differences [2,3,4,5,6]. This evolutionary background has served as a basis for several myths about cat nutrition. For example, sometimes the fact that cats are strict carnivores is interpreted as meaning that cats can only obtain their nutritional requirements through consuming animal tissue. This is incorrect from a nutritional perspective as animals, including cats, need nutrients and not specific ingredients [7]. The fact that cats evolved consuming low-carbohydrate prey and the increased understanding of the unique feline carbohydrate metabolism has led to speculations that high-carbohydrate diets could be detrimental to feline health. The most commonly expressed concern is that cats poorly digest and metabolise carbohydrates, while others warn that excess carbohydrates could lead to feline obesity and/or diabetes mellitus [4,5,8,9,10]. Therefore, researchers evaluated high-carbohydrate diets as a risk factor for the development of feline obesity and diabetes mellitus; others examined the use of low-carbohydrate diets for the prevention and treatment of these metabolic diseases.

## 2. Definition, Classification and Function of Dietary Carbohydrates

When discussing the benefits and adverse effects of dietary carbohydrates, one has to keep in mind the definition, classification and function of carbohydrates. Carbohydrates or saccharides (CH_2_O_n_) are biological molecules that are composed of carbon, hydrogen and oxygen [11].

### 2.1. Classification of Carbohydrates

**Figure 1:**
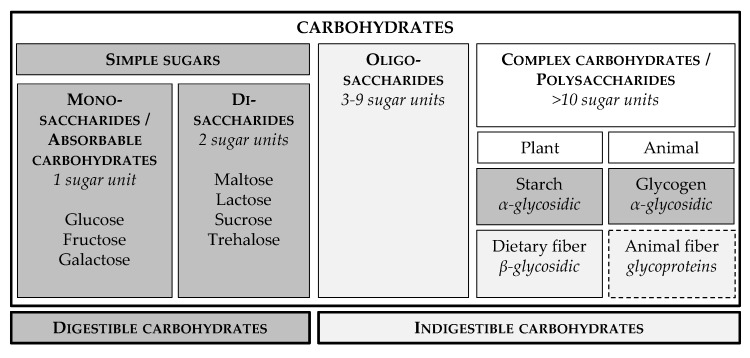
Classification of carbohydrates including simple sugars, oligosaccharides and complex carbohydrates. This literature review focusses especially on digestible carbohydrates, i.e., monosaccharides, disaccharides and starches.

Classification is based on chemical structure and degree of polymerisation and includes: monosaccharides, disaccharides, oligosaccharides and polysaccharides [11,12] (Figure 1). Monosaccharides (glucose, fructose and galactose) are also called absorbable carbohydrates as these compounds do not require enzymatic digestion and can be absorbed directly. Disaccharides (lactose, sucrose, maltose and trehalose) are readily digested by intestinal enzymes in most mammals [11,12,13]. The oligosaccharide group with three to nine sugar molecules is resistant to enzymatic digestion and is fermented by microbial enzymes in the large intestine [12,13]. The polysaccharides or complex carbohydrates, encompassing 10 or more sugar units, can be further defined based on the digestibility and the nature of the glycosidic bonds between the sugar units [12]. Starches are complex carbohydrates that have α-glycosidic bonds and are therefore enzymatically digested by α-amylase in the mammalian small intestine [11,12,13]. Starch is the most abundant digestible carbohydrate produced during photosynthesis and serves as energy storage in plants [11,12]. Similarly, glycogen, a glucose polymer linked with α-glycosidic bonds, functions as long-term energy storage in animals. Glycogen is present in most animal tissues, with the highest content in liver and skeletal muscle [12]. Dietary fibre, another class of complex carbohydrates, is composed of cell wall polysaccharides (cellulose, hemicelluloses and some pectins), non-cellulose polysaccharides (pectins, gums and mucilage) and structural non-polysaccharides (lignin). The sugar units of dietary fibre are linked by β-glycosidic bonds, so dietary fibre is not digestible in the small intestine. Most dietary fibres are fermentable in the large intestine, although because of the limited length of the feline colon, certain fibres, like cellulose and wheat bran, are not extensively fermented [11]. Additionally, animal fibre has been overlooked in the carnivore diet. Animal fibre consists of indigestible glycoprotein-rich material, such as bone, tendon, cartilage, skin, hair and feathers, and is a substrate for large intestinal microbial fermentation [14].

The term “dietary carbohydrates”, as used in this literature review, is referring specifically to digestible carbohydrates, especially simple sugars (mono- and disaccharides) and starches (Figure 1).

### 2.2. Glucose Metabolism and Homeostasis

While digestible carbohydrates are not essential dietary nutrients, carbohydrate in the form of glucose is physiologically essential. The brain, red blood cells, leukocytes and other specialised cell types in the renal medulla, testes and eyes, depend on glucose for their energy needs [15]. It is therefore critical for the body to maintain a glucose supply for these tissues. To maintain blood glucose within its strictly regulated concentration range (cat: 3.9–6.7 mmol/L [16]; human: 4–6 mmol/L [15]) during episodes in which glucose is not being absorbed from the gastrointestinal tract, the body is able to produce glucose by breakdown of body glycogen stores and by gluconeogenesis [15]. Energy is provided to the body as ATP via the glycolysis and the citric acid cycle [15]. Simple carbohydrates and starches also provide building blocks for all other carbohydrates, including lactose produced by the mammary gland, ribose needed for nucleic acid synthesis and the sugar residues found in glycoproteins, glycolipids and glycosaminoglycans in the body [12,15]. Glucose is also needed for the synthesis of non-essential amino acids [17] and vitamin C [18].

## 3. Carbohydrate Digestion and Absorption

Due to evolutionary pressures, cats developed several physiologic adaptations of carbohydrate digestion and absorption that reflect their true carnivorous nature [3,5].

### 3.1. Reduced Digestive Enzyme Capacity

**Figure 2:**
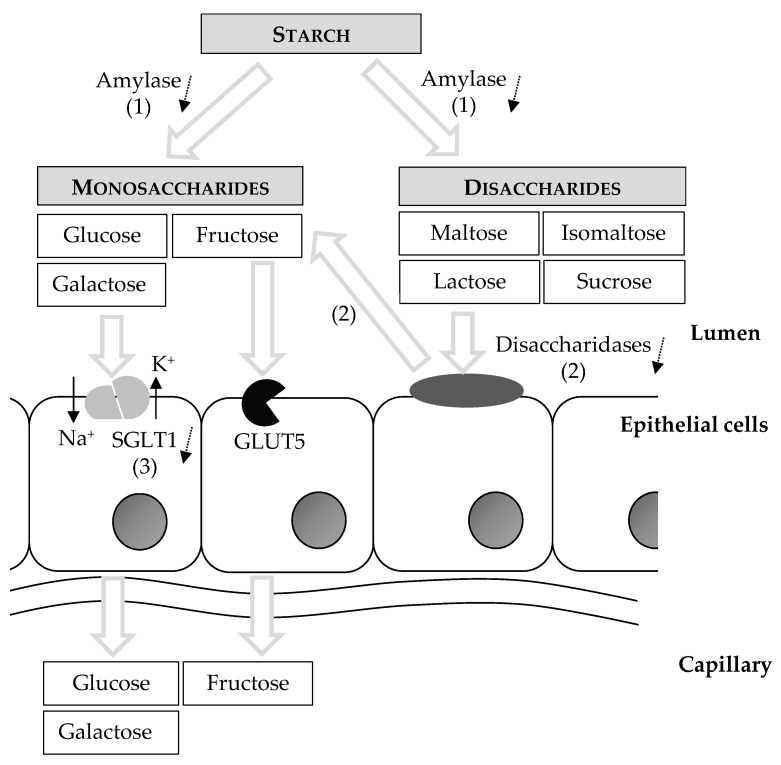
Starch digestion and absorption of monosaccharides in cats: (1) Cats have very limited salivary amylase and intestinal amylase activity is low; (2) adult cats have low disaccharidase activity in the small intestinal brush border; (3) the Na+/glucose co-transporter 1 (SGLT1) for active transport of glucose and galactose is expressed in cats, but the Na+-dependent glucose uptake is two times slower than in dogs and inability to upregulate SGLT1 has been suggested in cats.

Cats possess only a small capacity for starch digestion by endogenous intestinal enzymes (Figure 2). Similar to dogs [19], salivary amylase responsible for initiation of starch digestion in the feline saliva is very limited [20]. Amylase is found in the feline pancreas and chyme; however, the intestinal amylase activity is low compared to other animals [20,21]. In addition, pancreatic tissue in adult cats has low maltase activity, only traces of isomaltase and no lactase and sucrase activity. The disaccharidases (i.e., maltase, isomaltase and sucrase) are present in the feline small intestinal mucosa, but again the activity in the intestinal brush border is low compared to other species. Also, the distribution is peculiar as the disaccharidase activity increases in cats from cranial to caudal, while in other species, for example in dogs, the maximum occurs in the jejunum. Disaccharidase activity was not affected by dietary carbohydrate level or source (i.e., cooked corn starch, raw corn starch, raw potato starch, sucrose, lactose versus carbohydrate free), which supports an inability of cats to adapt enzyme activity to increased dietary carbohydrate concentrations [22].

### 3.2. Alterations of Monosaccharide Absorption

Once the starches are digested, monosaccharides are absorbed (Figure 2). Glucose and galactose are transported over the apical enterocyte membrane by Na+/Glucose Co-Transporter 1 (SGLT1). Absorption is coupled to Na+ and its electrochemical gradient provided by Na+/K+-ATPase activity [23]. The Na+-independent fructose transporter, GLUT5, transports fructose from the intestinal lumen into enterocytes down its concentration gradient [23]. Anatomically it may seem as cats have a limited ability to absorb monosaccharides because of the limited length of the small intestine. However, partial compensation for this relative deficit in absorptive surface occurs. First, the mucosal area per unit serosal area in the cat’s small intestine is greater compared to rats and dogs [24]. Second, the d-glucose transport across the feline intestinal brush border appears to have a considerably higher capacity in cats than in cattle and rabbits, despite the similar amounts of glucose reaching the small intestine when these species are fed a natural diet [25]. Although omnivorous species can adapt glucose transport to dietary carbohydrate intake, it was found in kittens that the glucose transport system in the intestinal brush border does not adapt to varying levels of dietary carbohydrate [26]. Still, this study had a very small study population and may not reflect the activity of the glucose transport system in adult cats. More recently it was found that cats express SGLT1 in the small intestine. In all small intestinal regions, the initial rate of Na+-dependent glucose uptake was approximately two times slower in cats compared to dogs [27]. Batchelor et al. also reported absence of expression of the T1R2 subunit of the sweet receptor in the feline intestine and explains that, as dietary regulation of intestinal SGLT1 expression involves sensing by the intestinal sweet receptor, the T1R2/T1R3 heterodimer, this absence may suggest inability to upregulate SGLT1 in response to dietary carbohydrates [27]. However, regulation of glucose transport by increasing or decreasing sugar concentrations was not investigated. Based on the current literature, it is impossible to conclude what concentration of sugars is needed after ingestion of carbohydrate-containing diets to reach a maximum rate of glucose transport over the intestinal brush border and if such concentration would be reached with current commercial cat food.

### 3.3. Carbohydrate Digestibility

Early studies by Morris et al. observed that adult cats could efficiently digest all carbohydrates added to a meat-based diet, despite the described evolutionary adaptations along the feline gastrointestinal tract [28]. The total apparent digestibility of starch is reported to be 40–100%, depending on source and treatment [28,29,30], which proves that cats can digest and absorb carbohydrates. As in other mammals, proper processing and cooking is necessary. Carbohydrate sources are not provided to cats as raw ingredients. Typically, carbohydrate sources are ground and cooked during the extrusion or canning process, which improves digestibility. Poorly digestible carbohydrates or excessive amounts of highly digestible carbohydrates that are not digested in the small intestine provide substrate for microbial fermentation in the colon. High carbohydrate intake in cats therefore increases colonic and faecal organic acid concentrations and reduces faecal pH [21]. Also, adverse digestive effects, such as diarrhoea, flatulence and bloating, may be induced [21].

## 4. Carbohydrate Metabolism

**Figure 3:**
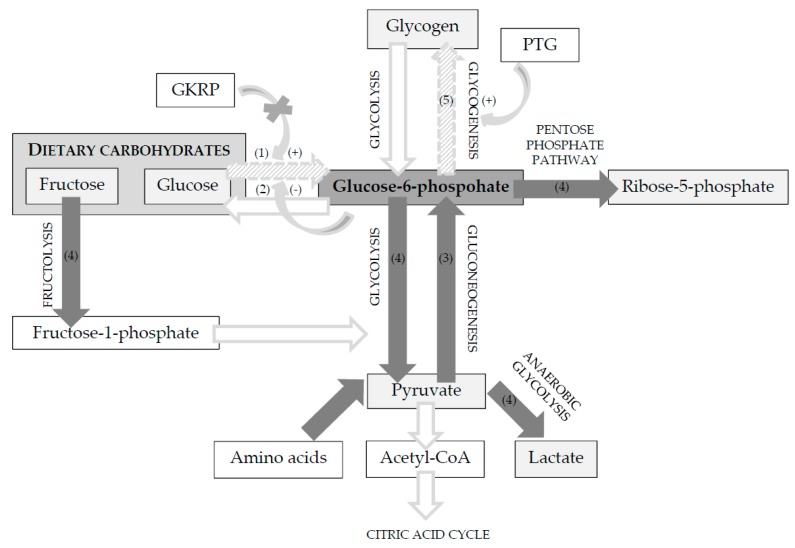
Glucose metabolism in feline hepatocytes. (1) Minimal to absent hepatic glucokinase activity [32,33,34,35], and no glucokinase gene expression [35]. No regulation of glucokinase by glucokinase regulatory protein (GKRP). Although the *GCKR* gene is present, *GCKR* mRNA expression and GKRP activity are absent [33,36]. (2) Upregulation of other hexokinases seems unlikely due to inhibition by glucose-6-phosphate, which is continuously formed by gluconeogenesis; (3) activities of rate limiting enzymes of gluconeogenesis (i.e., pyruvate carboxylase, fructose-1,6-biphosphatase, and glucose-6-phosphatase) are upregulated [34,35]; (4) glucose-6-phosphate-mediated inhibition of hexokinase I, II and III may be overcome by activation of glucose disposal pathways that occur distal to the glucokinase-mediated phosphorylation step, promoting glucose oxidation pathways such as glycolysis (i.e., pyruvate kinase), fructolysis (i.e., fructokinase), pentose phosphate pathway (i.e., glucose-6-phosphate dehydrogenase) and anaerobic glycolysis (i.e., lactate dehydrogenase) [35,37] and stimulate glycogenesis; (5) minimal activity of hepatic glycogen synthase has been assumed [32]. However, glycogen content in the feline liver is similar to in dogs and humans [39]. Activation of glycogenesis by protein targeting to glycogen (PTG) may allow synthesis of glycogen when glucose is lacking, using gluconeogenic precursors such as amino acids [38].

Carbohydrate metabolism is also altered by some major evolutionary adaptations in the enzyme activities of the feline liver [3,5]. Peculiarities of glucose metabolism in the feline liver are summarised in Figure 3.

### 4.1. Lack of Glucokinase Activity and Possible Compensation Mechanisms

In most species, glucokinase (or hexokinase IV) is responsible for the phosphorylation of glucose to glucose-6-phosphate, the first step of glycolysis. Glucokinase has a maximal rate of glucose phosphorylation and operates only when the liver receives a large load of glucose from the portal vein [15,31]. The hepatic glucokinase activity is regulated by the glucokinase regulatory protein (GKRP). When fasted, glucokinase and GKRP are bound and reside in the hepatocyte nucleus. In response to a meal, glucokinase translocates to the cytosol and becomes enzymatically active [15]. This regulatory mechanism enables the liver to respond to an increased demand for glucose phosphorylation. Several authors reported minimal to absent hepatic glucokinase activity in cats [32,33,34,35] and, according to Tanaka et al., no glucokinase gene expression was observed in the feline liver [35]. In contrast, dogs have higher glucokinase activities, similar to omnivores [32]. Although the *GCKR* gene that encodes GKRP is present in feline hepatocytes, *GCKR* mRNA expression and GKRP activity are absent [33,36]. More research is needed to understand how glucose phosphorylation can proceed efficiently in the absence of glucokinase.

It is often assumed that upregulation of other hexokinases [35,37], with a higher affinity for glucose [31], compensates for the lack of glucokinase activity in cats. Still, this was doubted by Schermerhorn as, unlike glucokinase, hexokinase I, II and III are inhibited by glucose-6-phosphate, which is continuously formed by gluconeogenesis in feline liver [38]. One plausible mechanism is activation of glucose disposal pathways that occur distal of the glucokinase-mediated phosphorylation step, promoting glycolysis and glycogen production [38]. When compared to dogs, cats have upregulated activities of enzymes involved in various glucose oxidation pathways (i.e., glycolysis (pyruvate kinase), fructolysis (fructokinase), pentose phosphate pathway (glucose-6-phosphate dehydrogenase) and anaerobic glycolysis (lactate dehydrogenase)) [35,37]. A limited ability to store glucose as glycogen due to minimal activity of hepatic glycogen synthase has been assumed in cats, as a study found low rates of incorporation of C^14^-labelled glucose in glycogen [32]. However, liver glycogen encompasses approximately 5% of the liver weight in cats, which is similar in dogs and humans [39]. Interestingly, in rat hepatocytes, overexpression of protein targeting to glycogen (PTG) stimulates glycogenesis, even in the absence of glucose [40,41]. No data are available in cats on PTG-mediated activation of glycogenesis, but activation of glycogenesis by PTG may allow feline hepatocytes to synthesise glycogen when glucose is lacking, using carbon originating from gluconeogenic precursors [38].

### 4.2. High Gluconeogenic Activity and Link with Protein Requirement

In feline livers, the activities of rate limiting enzymes of gluconeogenesis (i.e., pyruvate carboxylase, fructose-1,6-biphosphatase, and glucose-6-phosphatase) are higher than those in canine livers [34,35]. It was originally thought that, in contrast to omnivores, gluconeogenesis is continuously active in the feline liver, irrespective of the dietary protein content [42]. Nonetheless, Kettlehut et al. revealed increased gluconeogenesis during fasting when cats were fed a high-carbohydrate diet. When consuming a high-protein, low-carbohydrate diet, gluconeogenic capacity is already high or even higher in the postprandial state than during fasting [43]. Studies also showed that the magnitude of the gluconeogenesis is similar in cats and humans during fasting, but also when fed [41,44]. This demonstrates that in carnivores and omnivores gluconeogenesis is similarly important in the postprandial state and not only when fasted. To date, multiple studies have demonstrated that cats can adapt metabolically to variations in dietary macronutrients and regulate glucose and protein metabolism when necessary [41,44,45,46,47].

**Figure 4:**
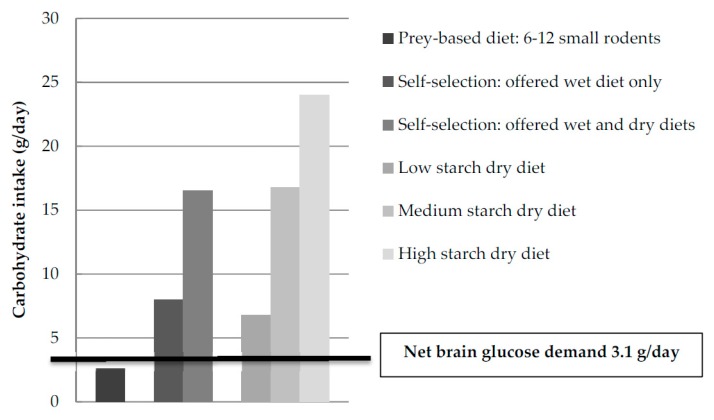
Carbohydrate intake following consumption of a prey-based diet (NFE 1–2%ME, carbohydrate intake presented is a calculated average of 6–12 small rodents per day including gut content, glycogen and glucose from glycerol) [1,48], following self-selection from only wet diets (NFE 12%ME) [50] or from diets with different texture and moisture content (NFE 11–21%ME [51], and following ingestion of dry diets with various starch levels (NFE 12%, 30% and 43%ME, respectively) [52] compared to the calculated net brain glucose demand [48].

Interestingly, Eisert proposed that cats, being hypercarnivores (i.e., small carnivorous mammals with a proportionally large brain), have a high brain glucose demand [48]. Using data from Kley et al. [44], Eisert estimated that the brain glucose demand of cats represents approximately 30% of gluconeogenesis in fasted cats [48]. The equivalent value for humans with a much larger brain is approximately 44% [48,49]. This high endogenous glucose demand of the brain as well as other obligate glucose-consuming tissues cannot be met by carbohydrates present in the natural prey-based diet including gut content, glycogen and glucose from glycerol [48] (Figure 4). Eisert noted that in cats a close agreement exists between the nitrogen loss predicted from brain glucose demand and the reported endogenous urinary nitrogen loss [48]. This explains a high capacity for de novo synthesis of glucose from amino acids and increased dietary protein and amino acid requirements in cats, which is discussed elsewhere [6].

Consequently, cats can digest carbohydrates and absorb and utilise glucose in a manner similar to other species. The presence/absence and kinetics of specific intestinal and hepatic enzymes are consistent with the cat’s natural eating behaviour. Wild cats eat small vertebrate prey with low carbohydrate content (NFE 1–2%ME) [1,48], and must catch 8–12 of these small rodents every 24 h to provide their energy requirement [53]. Similarly, domestic cats in an experimental setting also spread their daily intake of food and macronutrients over 12–20 small meals, evenly spread between light and dark periods [53,54]. Because of the limited intestinal enzyme capacity, consumption of excessive amounts of digestible carbohydrates will not lead to a massive increase of glucose absorption, but will provide substrate for microbial fermentation in the colon, and cause gastrointestinal adverse effects. Fast action of glucokinase is redundant as only small amounts of glucose enter the bloodstream at a given time. In spite of this, cats are not limited in their ability to phosphorylate and metabolise glucose. A compensation for the lack of glucokinase is assumed, yet further research is needed. The activities of the rate-limiting enzymes involved in glycolysis and other glucose oxidation pathways are greater than those found in dogs, and cats, with the exception of glucokinase, have an abundance of other hexokinases.

Normal glucose metabolism in domestic cats seems to overlap with diabetes pathology in non-carnivores [38]. Glucokinase deficiency in rodents (transgenic and gene knockout studies) and humans (mature onset diabetes of the young type 2, MODY2) is characterised by hyperglycemia due to impaired hepatic glycogen synthesis and results in diabetes mellitus [55]. Although healthy cats lack glucokinase, liver glycogen content is similar in cats and humans [41], as feline liver glycogen most likely originates from other gluconeogenic substrates rather than glucose [38]. Also, healthy cats do not show persistent hyperglycaemia. Lean and obese cats are able to maintain glucose homeostasis over a seven-day period and daily glucose fluctuations are small. Fasting glucose concentrations in cats are also not different from those of humans and other mammals [16].

## 5. Taste, Food Selection and Macronutrient Balancing

### 5.1. Feline Sweet Blindness

Cats do not prefer foods with a sweet taste. In a two-choice preference test, cats are unable to distinguish between pure water and sucrose dissolved in water [56,57,58]. Neurophysiologic studies of the facial nerve demonstrated responses to salt, bitter, sour, amino acids and nucleotides taste stimuli [59,60]. No neural responses to sucrose and other sugars were detected in cats [61,62]. The sense of taste in cats is therefore similar to that of other mammals, with the exception of insensitivity to sweeteners. The molecular basis for this feline sweet blindness is the lack of a sensory system to detect sweet stimuli. Li et al. characterised the sweet receptor genes of cats and concluded that *Tas1r3* is expressed and functional, but *Tas1r2* is an unexpressed pseudogene. The functional sweet receptor heterodimer (T1R2/T1R3) can therefore not form, resulting in an inability to taste sweet taste stimuli [63,64]. This was confirmed in lions. Lions also possess the pseudogenized *Tas1r2* and show no preference for any of the natural sugars and artificial sweeteners tested [65].

### 5.2. Nutritional Geometry

Originally, Cook et al. concluded that kittens did not regulate their protein intake in a consistent manner as they did not eat more of a high-protein food compare to a low-protein food when offered a choice [66]. More recently, nutritional geometry has allowed researchers to investigate the complex interactions among dietary protein, fat and carbohydrates [50,51,67]. After reassessing the data of the study by Cook et al. [66] the conclusion seemed inaccurate [50]. In a controlled environment cats are able to regulate their macronutrient intake when provided with a choice of dry foods or wet foods and showed a target macronutrient intake [50] (Table 1). Interestingly, this selected macronutrient profile is very similar to the reported macronutrient composition of the diet of free-roaming feral cats [1] (Table 1).

In a first study by Hewson-Hughes et al., only cats offered wet test foods reached the target intake with a single diet. This was not possible with the dry test foods due to macronutrient composition of these diets, yet cats altered the proportions of different experimental dry foods to approach the intake target as close as possible to the target nutrient balance [50]. A second study confirmed the ability of cats to balance macronutrient intake by food selection when offered a variety of diets that not only differed in macronutrient content, but also had different moisture content, texture and energy density [51] (Table 1). It was also shown that when testing diets to which flavours of different attractiveness (i.e., fish, rabbit and orange), were added, diet selection was independent of organoleptic properties. The authors concluded that, although flavour and aroma may influence diet selection in the short term, over time, macronutrient balancing is the primary driver of diet selection and macronutrient intake in cats [67]. It is, however, impossible to compare the selected macronutrient profiles of this study with previous studies as all test diets had negligible to low carbohydrate content (NFE 0–3%ME).

The nutritional geometry studies showed that domestic cats specifically aim for a high intake of protein, which is limited with high-fat and high-carbohydrate diets. When dietary protein intake was low, fat intake to achieve balance was flexible and cats would eat more of the high-fat food to reach target protein intake. For carbohydrate, there was an absolute constraint that limited further food intake and resulted in deficits relative to the target of both energy and protein if consuming a low protein diet [50]. As a result, a carbohydrate intake ceiling of about 300 kJ per day was proposed [50,51]. It was explained that this carbohydrate ceiling could be reflective of the metabolic adaptations along the feline gastrointestinal tracts (see Section 3 above). Still, Figure 4 shows daily carbohydrate intakes similar to what domestic cats select [50,51], when consuming extruded cat foods with low (NFE 11%ME) or medium (NFE 30%ME) starch levels [52]. Yet, the calculated amounts of digestible carbohydrates in a prey-based diet are lower [48].

## 6. Carbohydrates in Pet Food

### 6.1. Conventional Dry and Canned Pet Foods

The majority of cat owners feed their cats conventional pet food because it is convenient and economical [68,69]. Many cats are offered dry food but, compared to dogs, cats are more likely to receive moist food as part of their diet [68]. These conventional cat foods can contain up to 55%ME as carbohydrates, allowing for a minimum protein (25%ME) and fat (20%ME) content as set by the American Association of Feed Control Officials and The European Pet Food Industry Federation [70,71]. However, most commonly conventional cat foods provide between 20% and 40%ME as carbohydrates [72]. Carbohydrate ingredients, such as grains, potatoes, legumes, etc., composed primarily of starches, are important for pet food processing. A certain level of starch must be included for proper processing of dry product. The main function of carbohydrates in the processing of extruded dry diets is to provide structural integrity to the kibble. Dry food cannot hold its form or structure without the binding capacities of carbohydrates. It is cooked, gelatinised starch that binds the kibble together and prevents crumbling [73]. The carbohydrate–protein interactions that occur also contribute to texture and flavour [74]. Although moist foods contain a relatively smaller proportion of digestible carbohydrates compared to dry food [30,72], processing characteristics of moist foods are also affected by carbohydrates. Most moist diets contain gelling agents, which are usually carbohydrates that form a gel upon processing [75]. Starches gelatinise with denaturing protein to give the loaf structure, maintaining even distribution of the formulation. The textural characteristics also vary widely among carbohydrate sources because each source reacts uniquely to temperature and time [73].

### 6.2. Alternative Pet Diets

Over the last decades, the use of alternative diets such as bone and raw food, raw meat-based diets, home-cooked or commercially produced fresh meat diets as well as vegetarian and vegan diets [76,77,78] has become more popular. A survey showed that 9.6% of cats received bones and raw food as part of their main meal [68]. These raw food diets are often used by owners who want to feed their cats a more natural diet (i.e., minimal processing and less grain content) [79]. Those types of alternative diets typically contain a lower amount of carbohydrates compared to traditional commercial diets. Advocates believe these alternative diets have several health benefits, while those opposed focus on the risks and potential complications. There is a lack of large cohort studies evaluating these purported benefits and risks. One recent study showed a better apparent digestibility of dry matter, organic matter, crude protein and gross energy in kittens fed a raw diet compared to heat-processed diets [80]. On the other hand, evidence exists that raw meat-based diets can be contaminated with pathogens (e.g., *Salmonella*, *E. coli*, *Campylobacter*). Pets exposed to these pathogens can exhibit clinical signs or can be clinically normal and shed the bacteria in faeces [81]. These pathogens not only bear a risk for the pet but also for humans sharing the same environment [81,82]. These types of diets also pose other health risks like nutritional imbalances mainly for minerals, trace elements and vitamins; increased risk for gastrointestinal obstruction/penetration by bone fragments; and possible exogenous thyrotoxicosis due to contamination with raw thyroid tissue [76,83,84,85]. However, these concerns are often based on isolated case reports [82].

There is much debate about the appropriate amount of carbohydrates in cat food. As in many other mammals, there is no minimal dietary carbohydrate requirement for cats [11]. Many traditional commercial diets contain more carbohydrates than a feral cat would consume or domestic cats prefer when they have the choice (Figure 4). These data, however, do not show what carbohydrate level is optimal for feline health. Moreover, lifestyle differences (e.g., spay/neuter status, indoor versus outdoor lifestyle, etc.) between domestic and wild cats may also affect optimal dietary nutrient content.

## 7. Energy Efficiency, Weight Gain and Obesity

To date, more than 50% of cats in industrialised countries are overweight or obese [86,87]. Although it is often assumed that feline obesity is on the rise, a relatively constant distribution of body condition was noted in a specific cat population over a four-year timespan [88,89]. Also, cat households surveyed in the same city in 1993 and 2007 did not show a difference of obesity prevalence [86,90]. Still, obesity is noted as the number one nutritional problem in cats and represents a major health risk for the feline population. Obesity predisposes cats to devastating health complications such as insulin resistance, diabetes mellitus, lower urinary tract disease, osteoarthritis and skin problems [91,92,93]. Although no data are available in cats, reduced quality of life [91] and shortened life span [94] were observed in dogs with higher caloric intake.

### 7.1. Risk Factors of Feline Obesity

Many risk factors affect the balance of energy intake and energy utilisation. A positive energy balance is created when the intake of calories is in excess of the cat’s needs and results in fat deposition and consequently weight gain [91,92,93]. When asked, veterinarians felt that only 3% of dog obesity cases were attributed to animal-specific factors, while 97% was caused by factors that were controlled by the owner (i.e., diet, exercise and household characteristics) [95]. Predisposing factors for feline obesity are summarised in Table 2. Recently, Pretlow and Corbee [96] described resemblances between childhood and pet obesity as both pet-parents and child-parents try to obtain affection by giving treats and/or large meals. Dietary factors include highly palatable, high-energy diets, unrestricted access to food [97,98,99], and inclusion of homemade food, table scraps and/or treats [98]. Most studies found no influence of commercial diet types on the prevalence of obesity [69,86,90,100]. However, some reported a higher risk in cats consuming “premium” and/or dry foods [89,101,102]. These epidemiological studies did not assess dietary macronutrient content and food intake, so no conclusions can be drawn regarding dietary carbohydrate or fat content and/or intake of these macronutrients. Higher energy density of “premium” pet foods compared to most “economy-type” dry diets and dry food compared to wet food is a plausible explanation. Owners tend to feed dry food free choice [88] and therefore, excess energy consumption happens easily. When cats were fed ad libitum, a wet diet induced a decrease in voluntary energy intake and body weight compared to the freeze-dried version of the same wet diet [103]. Also adding water to dry food without changing energy intake or dietary macronutrient content promoted physical activity and prevented body weight gain [104,105,106]. Still, Thomas et al. was not able to reproduce an effect of dietary moisture on physical activity when cats were kept outdoors, but observed a stronger diurnal activity pattern when cats were fed dry food [107]. Despite an increased proportion of owners feeding dry food, Australian researchers found no association with dry food feeding in 1993 [90] or 2007 [86] and no increase of obesity occurred over this period [86].

### 7.2. Macronutrients and Weight Gain

Because of the discrepancy in carbohydrate content between a natural prey diet and currently available traditional commercial cat foods, excess carbohydrate intake is often considered the primary cause of feline obesity [4,8,9]. It has been postulated that excessive amounts of dietary carbohydrates cause an overproduction of insulin resulting in excess fat deposition [4,9]. Similar to nitrogen balance, glucose balance is strictly controlled. It is well established in mammals that increased carbohydrate ingestion enhances glycogen reserves. Still, the maximum capacity of glycogen storage is small and enlargement of these stores stimulates glucose oxidation. In humans, in contrast to common belief, the conversion of carbohydrates to body fat is low, even when ingested in large amounts. Fat oxidation is not affected as much by increased fat intake and dietary fat is stored very efficiently as body fat [123]. In fact, Nguyen et al. and Backus et al. confirmed increased weight gain and expansion of fat mass when cats had free choice access to a low-carbohydrate, high-fat diet (NFE 28%ME, fat 40%ME [124]; NFE 3%ME, fat 64%ME [125]) in comparison to a high-carbohydrate, low-fat diet (NFE 42%ME, fat 27%ME [124]; NFE 57%ME, fat 9%ME [125]) [124,125]. This is not surprising as fat contains twice as much energy per gram compared to carbohydrates and protein and thus high-fat diets have a higher energy density. Additionally, fat improves palatability and promotes energy intake when offered free choice. Coradini et al. also concluded that a low-carbohydrate, high-protein diet (NFE 23%ME, protein 47%ME) resulted in increased fat deposition and greater weight gain when fed ad libitum compared to a high-carbohydrate, low-protein diet (NFE 51%ME, protein 21%ME) with similar energy density. It is important to note that commercially available cat foods with variable ingredient composition were used for this study, and the findings could be due to a slightly higher proportion of fat in the low-carbohydrate, high-protein diet or because cats limit their carbohydrate intake and hence energy consumption when consuming high-carbohydrate diets [50,126]. The latter possibly is less likely since cats fed the high-carbohydrate diet (NFE 51%ME) consumed twice as many carbohydrates compared to the carbohydrate amount self-selected by domestic cats (NFE 12%ME) [50], although the greater carbohydrate consumption may also reflect a longer adaptation period to the high-carbohydrate diet (12 weeks [126] versus one week [50]). These diets may also have varied in nutrient bioavailability, especially as loose stool was observed in cats fed the high-carbohydrate, low-protein diet [126].

Based on the research discussed above, carbohydrates do not appear to be the biggest concern in the development of obesity. Overfeeding and thereby consumption of excess calories of any macronutrient is a much more important risk factor for obesity and should be the main focus of obesity prevention. There is a need to improve owner education with a focus on feeding a measured amount of food appropriate for each cat’s individual energy requirements and accounting for factors such as spay/neuter status, aging, and inactive lifestyle, which reduce the energy requirement. Attention should also be paid to feeding strategies that provide environmental enrichment and increase physical activity.

## 8. Diabetes Mellitus, Insulin Sensitivity and Postprandial Glycaemia

Diabetes mellitus is a common disease in cats. The majority of cats develop type 2 diabetes mellitus, which results from insulin resistance and β-cell dysfunction. The pathophysiology of this disease resembles the disease in humans [127,128,129,130]. The prevalence, estimated at 0.20–1.25% [111,112,114,117,118,119], seems to be on the rise [118]. The increase between 1970 and 1999 may reflect an increase in the willingness of cat owners to seek advanced veterinary medical care or may be due to an increase in the prevalence of major risk factors associated with the disease [118]. No studies have investigated time trends of feline diabetes in the 21st century.

### 8.1. Risk Factors of Feline Diabetes Mellitus

Risk factors associated with feline diabetes mellitus are summarised in Table 2. The effect of diet type remains a topic of debate. Slingerland et al. failed to find evidence that the energy percentage of dry food in the cat’s diet is a risk factor for the development of feline diabetes [121]. Also, in the study by Sallander et al. healthy controls consumed a higher portion of dry food compared to diabetic cases [117] and McCann et al. observed that cats eating a combination of dry and wet food are at higher risk compared to cats fed only dry or only wet food [111]. More recently, dry food feeding has been associated with increased risk of diabetes mellitus in normal-weight cats, while diet type was not found to be a risk factor in obese cats [110]. Body condition was, however, reported by the pet owners in this study, which may have skewed the body condition classification, as previous research has shown that pet owners underestimate their cat’s body condition [86,90,98,100,108]. As with the epidemiological research on risk factors for obesity, dietary macronutrient content and food intake were not assessed, so conclusions relating to dietary carbohydrate content, which are often made in these publications, cannot be drawn. Other dietary factors increasing the risk for diabetes include greedy eating behaviour and free choice feeding [110], which could be linked to higher energy intake and obesity. Obese cats are up to four times more likely to develop diabetes mellitus [110,111,114,117,118,119,122] as obesity is associated with a reduction in insulin sensitivity [131,132,133,134]. Hoenig et al. demonstrated that insulin resistance and decreased glucose effectiveness in cats were caused by obesity but not by dietary protein or carbohydrate content. Each kilogram increase in body weight led to a 30% reduction in insulin sensitivity and glucose effectiveness and weight loss normalised insulin sensitivity [47].

### 8.2. The Carbohydrate-Diabetes Hypothesis

Dietary carbohydrates are often postulated to increase the risk of diabetes mellitus in cats [9,10]. It is hypothesised that consumption of excessive amounts of highly refined and easily digestible carbohydrates places a large demand on the pancreatic β-cells for excessive insulin secretion, which may eventually result in amyloid deposition, β-cell exhaustion, and ultimately loss of insulin-producing β-cell and diabetes mellitus [10]. This hypothesis is based on the theory that cats have a limited ability to process large glucose loads due to diminished glucose sensing (see Section 4).

### 8.3. Response to Intravenous Glucose and Oral Carbohydrate Administration

Cats release insulin very rapidly in response to intravenous glucose administration [131,132,134,135]. However, Curry et al. concluded that feline pancreatic β-cells are less sensitive to glucose than those of omnivores, and amino acids seem to be more potent modulators of pancreatic insulin release [136]. Intravenous glucose tolerance tests (IVGTT) have shown that, despite a rapid response to glucose, glucose clearance is delayed in cats compared to other animals. After intravenous administration of 1 g glucose/kg body weight, blood glucose concentrations return to baseline at 90 min in cats [131], at 40–60 min in dogs [137] and at 30–40 min in humans [138]. Chronic hyperglycaemia (30 mmol/L) induced by 10-day intravenous glucose infusion has been shown to impair insulin secretion, decrease β-cell number and induce diabetes keto-acidosis in cats [139]. The glucose concentrations observed in this study are consistent with uncontrolled diabetes, yet this is not a physiological situation that occurs with food intake (Table 3). Intravenous tests bypass the gastrointestinal tract and do not account for the incretin effects on insulin response.

Postprandial glucose concentration and insulin response depend on the type of carbohydrates added to the diet. Oral administration of 2 g glucose/kg body weight is followed by a prompt increase in glucose and insulin [141] (Table 3). Experimental diets containing up to 34%ME of glucose induced a steep rise in blood glucose 1 h after feeding (Table 3). Also, glucosuria was observed with consumption of diets containing simple sugars, especially glucose and galactose [142]. Still, in contrast to processed foods sold for human consumption, commercial cat food contains very limited amounts of simple sugars. Most of the carbohydrates found in commercial pet foods are in the form of complex carbohydrates, including starches and fibre [8]. Eleven hours after a single high-starch meal (NFE 43%ME), plasma glucose concentrations increased and remained high, while in dogs this increase occurred after 7 h. When cats and dogs were fed a low- (NFE 11%ME) or medium-starch (NFE 30%ME) diet, this postprandial rise in glucose did not occur [52]. It is worth noting that other researchers did not observe hyperglycaemia and glucosuria when cats were fed a high-starch diet [30,142,144]. Moreover, the postprandial glucose concentrations reported are far below the level found in untreated feline diabetes [139] and are also below the definition for post-meal hyperglycaemia in humans [140] (Table 3).

In addition, different starch sources evoke different postprandial glucose and insulin responses due to variances in chemical composition of the starch sources and the diets [30,145]. For example in cats, a corn-based extruded diet evoked a higher glucose and insulin response compared to a lentil-based diet [30]. However, the differences are not as pronounced in cats as in dogs [146]. The postprandial glucose peak is delayed or may be subtle and the insulin response is less prominent in cats consuming dry foods with a variety of starch sources [30].

More recent research tested the postprandial glucose and insulin response in both cats and dogs following a single low-carbohydrate, high-protein test meal (NFE 7%ME, protein 64%ME) with or without added glucose (2 g/kg body weight) [143]. The postprandial glucose peak occurred later in cats (120 min) than in dogs (60 min), which reflects the adaptations of feline digestive enzymes and absorptive capacity. Also, the return to baseline glucose is delayed in cats (240 min) versus dogs (90 min) and the insulin response is not as prominent, which could be associated with the lack of hepatic glucokinase in cats [143]. A higher postprandial glucose response was noted when a baseline diet was loaded with 5 g/kg body weight of glucose or maltose compared to trehalose; corn starch had an intermediate response [147].

### 8.4. Macronutrients and Glucose Tolerance

Encouraged by the carbohydrate–diabetes hypothesis, a plethora of studies have investigated the effect of macronutrient levels on glucose and insulin metabolism using different methodology. Most of these studies are summarised by Verbrugghe et al. [5]. Briefly, Backus et al. and Thiess et al. demonstrated impaired glucose tolerance when feeding a low carbohydrate diet but attributed this to increased fat intake [125,144]. The two test diets in both studies differed only by substituting fat for carbohydrates (NFE 3%ME, fat 64%ME versus NFE 57%ME, fat 9%ME [125]; NFE 10%ME, fat 51%ME versus NFE 33%ME, fat 33%ME [144]). When carbohydrates were substituted for protein, a high carbohydrate diet (NFE 51%ME, protein 21%ME) was found to increase postprandial glycaemia and insulinaemia, compared to a low-carbohydrate diet (NFE 23%ME, protein 47%ME) [126]. Yet dietary protein and carbohydrate content were not found to affect insulin resistance and glucose effectiveness assessed with euglycaemic hyperinsulinaemic clamp [47]. In order to prevent confounding effects, isoenergetic substitution of the three energy sources (i.e., protein, fat and carbohydrates), is warranted. This results in three diets that differ by only one energy source, enabling identification of the effect of each specific energy source on glucose and insulin response. In healthy, non-obese cats, this three-diet strategy revealed no better glucose tolerance and insulin response to IVGTT when feeding a very low carbohydrate diet (NFE 7%ME, protein 45%ME, fat 48%ME), similar to the natural feline diet, compared to two diets with about 25–30%ME carbohydrates; a low-protein diet (NFE 29%ME, protein 28%ME, fat 43%ME) and a low-fat diet (NFE 25%ME, protein 47%ME, fat 27%ME) [131]. Farrow et al. demonstrated that feeding healthy, normal-weight cats a high-carbohydrate diet (NFE 48%ME, protein 25%ME, fat 26%ME) resulted in increased postprandial glycaemia when compared to two diets that both contained approximately 25–30%ME carbohydrates; a high protein (NFE 27%ME, protein 46%ME, fat 26%ME) and a high fat diet (NFE 26%ME, protein 26%ME, fat 47%ME) [148]. The high-carbohydrate diet contained more carbohydrates (48%ME) than common conventional cat foods (20–40%ME) [72]. Diets were fed free choice and although the energy intake was lower with the high-carbohydrate diet, the carbohydrate intake was still higher [148].

Although the research evidence regarding carbohydrates as a risk factor or cause of diabetes mellitus described above is limited, it does not seem that dietary carbohydrate intake is directly linked to feline diabetes mellitus. Obesity, due to inactive lifestyle and excessive caloric intake, and advancing age remain the greatest risk factors for diabetes mellitus.

### 8.5. Low-Carbohydrate Diets as Diabetes Treatment

The role of carbohydrates in the development and treatment of diabetes mellitus is not necessarily the same. In diabetes mellitus, simple sugars should be avoided at all times, as these are easily digested and readily absorbed. There is much more debate about the place of complex carbohydrates, especially starches, in the treatment of diabetes. Beneficial effects of feeding low-carbohydrate diets in the management of feline diabetes mellitus have been reported in several clinical studies. Frank et al. evaluated the effects of a low-carbohydrate diet (NFE 7%ME) in nine diabetic cats initially fed a high-fibre diet (NFE 32%ME). The daily insulin dose was reduced by over 50% in all but one cat, without loss of glycaemic control, three months after changing the diet [149]. Evaluating the effect of an α-glucosidase inhibitor (acarbose), combined with a low-carbohydrate diet (NFE 5%ME) in 18 diabetic cats, Mazzaferro et al. also found a decreased exogenous insulin dependence and improved glycaemic control when fed the low-carbohydrate diet and exogenous insulin therapy could be discontinued in 11 cats that were initially obese [150]. Similarly, 63 diabetic cats were randomly assigned to a moderate-carbohydrate, high-fibre diet (NFE 26%ME) or a low-carbohydrate, low-fibre diet (NFE 12%ME). Cats fed the low-carbohydrate, low-fibre diet were more likely to be well-regulated or to revert to a non-insulin-dependent state by week 16 when compared to the moderate-carbohydrate, high-fibre diet [151]. These studies suggest a beneficial effect of low-carbohydrate diets for the management of feline diabetes mellitus. However, Hall et al. reported no differences in clinical signs, insulin doses and peak and nadir blood glucose concentrations in cats fed a low-carbohydrate diet (NFE: wet food 10%ME, dry food 13%ME) compared to a maintenance diet (NFE wet food 3%ME, dry food 32%ME). Two of 12 cats achieved complete remission by the end of the study, with no differences between diets [152]. Still, it is noteworthy that the wet maintenance diet in this study was lower in carbohydrates than both low-carbohydrate test diets.

According to the ISFM Consensus Guidelines on Practical Management of Diabetes Mellitus in Cats, low-carbohydrate diets designed to manage feline diabetes are the preferred option [153]. Research evidence supporting this recommendation is limited and conflicting, and no randomised, controlled, blinded clinical studies have been performed. Published research includes a small number of cats and the test diets differed not only in carbohydrate content, but also in protein, fat and fibre content. Additionally, the ingredients were not standardised in the test diets, and they contained various carbohydrate and fibre sources, which may have affected glycaemic control.

Individualised diet therapy is recommended in diabetic cats. Although low-carbohydrate diets are a good option for some cats, good control and possibly remission can also be achieved with a higher carbohydrate diet designed for the management of diabetes. These higher carbohydrate diets do not contain simple sugars, have sources of complex carbohydrates that are less glycaemic and also include dietary fibre to help manage body weight and blood glucose response. Weight loss is a priority in obese diabetic cats, and can be accomplished using a low-fat, high-fibre veterinary therapeutic weight loss diet.

## Figures and Tables

**Table 1:** Energy distribution of a feral cat diet [1] and the diet selected by the adult domestic cats when offered only wet foods [50] or a combination of diets with different texture and moisture content [51].

	**Feral Cat**	**Domestic Cat**				
	Nutrient profile based on dietary habits and compositional data of consumed prey species [1]	Nutrient profile selected when offered wet diet only [50]	Nutrient profile when offered diets with different texture and moisture content [51]			
		3 wet	1 wet 3 dry	3 wet 1 dry	3 wet 3 dry	1 wet 1 dry
**Daily Energy Intake (%ME) from:**						
Crude protein	52%	52%	46%	44%	42%	48%
Crude fat	46%	36%	39%	35%	38%	41%
Nitrogen-free extract	2%	12%	15%	21%	20%	11%

**Table 2:** Animal, lifestyle and dietary factors increasing the risk for feline obesity and diabetes mellitus.

Factors Predisposing Cats to		
Obesity		Diabetes Mellitus
**Animal factors**		
Mixed breed [69,89,101,108] Norwegian Forest, British Shorthair, Persian [109]	**Breed**	Tonkinese, Norwegian Forest, Burmese Russian Blue, and Abyssinian [110,111,112,113,114,115]
Middle age [69,89,101,108,116]	**Age**	Older than 7 years [112,113,114,115,117,118,119]
Male [69,89,101,108,116,120]	**Gender**	Male [102,110,111,112,113,115,117,118,119,120]
Neutered [69,89,101,108,116]	**Sexual status**	Neutered [111,118,119]
**Lifestyle factors**		
Inactive cat [69,89,90,102]	**Physical activity**	Inactive cat [110,111,117,121]
Indoor confinement [69,89,102]	**Housing**	Indoor confinement [110,121]
**Dietary factors**		
Dry food: No influence [69,86,90,100] Increased risk [89,101,102]	**Diet type**	Dry food: No influence [121] Reduced risk [117] Increased risk [110]
Free choice feeding: Increased risk [97,99] No influence [69,86,89,90,108]	**Feeding method**	Free choice feeding and greedy eating behaviour [110]
Homemade food, human foods and/or treats [98]	**Other dietary inclusions**	
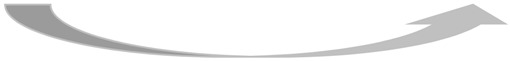		
**Obese cats are up to four times more likely to develop diabetes mellitus** [110,111,113,117,118,119,122]		

**Table 3:** Blood glucose response in healthy normal-weight cats following administration of IV glucose, oral glucose, and diets with various carbohydrate levels. Blood glucose concentrations are compared to normal glucose homeostasis in cats [16], concentrations causing β-cell dysfunction and β-cell loss in cats [139] and the definition of post-meal hyperglycaemia in humans according to the International Diabetes Federation [140].

Cats:	Intraday glucose fluctuations are small, glucose homeostasis is maintained within a strictly regulated concentration range of **3.9–6.7 mmol/L** [16]	
	IV glucose for 10d, clamp blood glucose at **25–30 mmol/L** (=untreated diabetes mellitus) leads to B-cell dysfunction and β-cell loss [139]	
Humans:	Post-meal hyperglycaemia = plasma glucose concentration **>7.8 mmol/L** 2 h after meal [140]	
**Carbohydrate Administration**		**Glucose Concentration**
IV 1 g/kgBW glucose [132]		- Peak at 5 min: **38.9 mmol/L** - Return to baseline at 90 min
Oral 2 g/kgBW glucose [141]		- Peak at 30 min: **7.8 mmol/L** - Return to baseline at 120 min
High glucose diet [142]: Glucose: 34%ME, 5 g/kgBW/day		- 60 min: increase **5.1 mmol/L** - 120 min: not measured - 180 min: not different from baseline
Glucose-loaded meal [143]: 13 g/kg BW high protein test meal (protein 64%ME, NFE 7%ME) +2 g/kgBW glucose		- Peak at 120 min: **10.8 mmol/L** - Return to baseline at 240 min
Diets with various starch sources [142] NFE content/intake: - Raw potato: 34%ME, 8.9 g/kgBW/d - Raw corn: 32%ME, 8.8 g/kgBW/d - Cooked corn: 27%ME, 4.7 g/kgBW/d		- Not different from baseline at any time: **3.65 mmol/L**
Extruded diets with the same starch source, but various starch levels [52] NFE content/intake: - LS diet: 11%ME, 1.7 g/kgBW/d - MS diet: 30%ME, 4.2 g/kgBW/d - HS diet: 43%ME, 6.0 g/kgBW/d		- LS: decreased 3–7 h post-meal - MS: no postprandial changes - HS: increased from 11 h post-meal, remained high (measured until 19 h): **6.9 mmol/L**
